# Updated evidence on cardiovascular and renal effects of GLP-1 receptor agonists and combination therapy with SGLT2 inhibitors and finerenone: a narrative review and perspectives

**DOI:** 10.1186/s12933-024-02500-y

**Published:** 2024-11-15

**Authors:** Kosuke Sawami, Atsushi Tanaka, Koichi Node

**Affiliations:** 1 Department of Cardiovascular Medicine, Graduate School of Medicine, The University of Tokyo, Tokyo, Japan; 2 Department of Cardiovascular Medicine, Saga University, 5-1-1 Nabeshima, Saga, 849-8501 Japan

**Keywords:** Glucagon-like peptide-1 receptor agonist, Sodium-glucose cotransporter 2 inhibitor, Finerenone, Combination therapy, Cardiovascular and kidney outcomes

## Abstract

Glucagon-like peptide-1 receptor agonists (GLP-1RAs) have a reliable hypoglycaemic and weight-loss effect that can intervene in obesity, which is the basis of type 2 diabetes pathology. GLP-1RA therapy has shown potential benefits in reducing the risk of major adverse cardiovascular events and improving kidney outcomes in patients with diabetes at high risk for cardiovascular disease. More recent evidence is expanding their benefits to heart failure with preserved ejection fraction and clinically important renal outcomes in patients with and without diabetes. Some sub-analyses of large clinical trials suggest that GLP-1RA and sodium-glucose cotransporter 2 inhibitor combination therapy may provide more significant reductions in heart failure hospitalization and renal composite events than each alone. Moreover, the addition of finerenone to this combination therapy could potentially provide stronger cardiorenal protective benefits. Further studies are needed to assess the potential cardiovascular and renal benefits of combination therapy and to determine suitable patient population for the therapy.

**Graphical abstract:**

Suspected clinical impacts of GLP-1RAs, SGLT2is, and finerenone oncardiovascular and kidney outcomes. CV, cardiovascular; GLP-1RA, glucagon-like peptide-1 receptor agonist; HFpEF, heart failure with preserved ejection fraction; HFrEF, heart failure with reduced ejection fraction; MI, myocardial infarction; SGLT2i, sodium-glucose cotransporter 2 inhibitor.
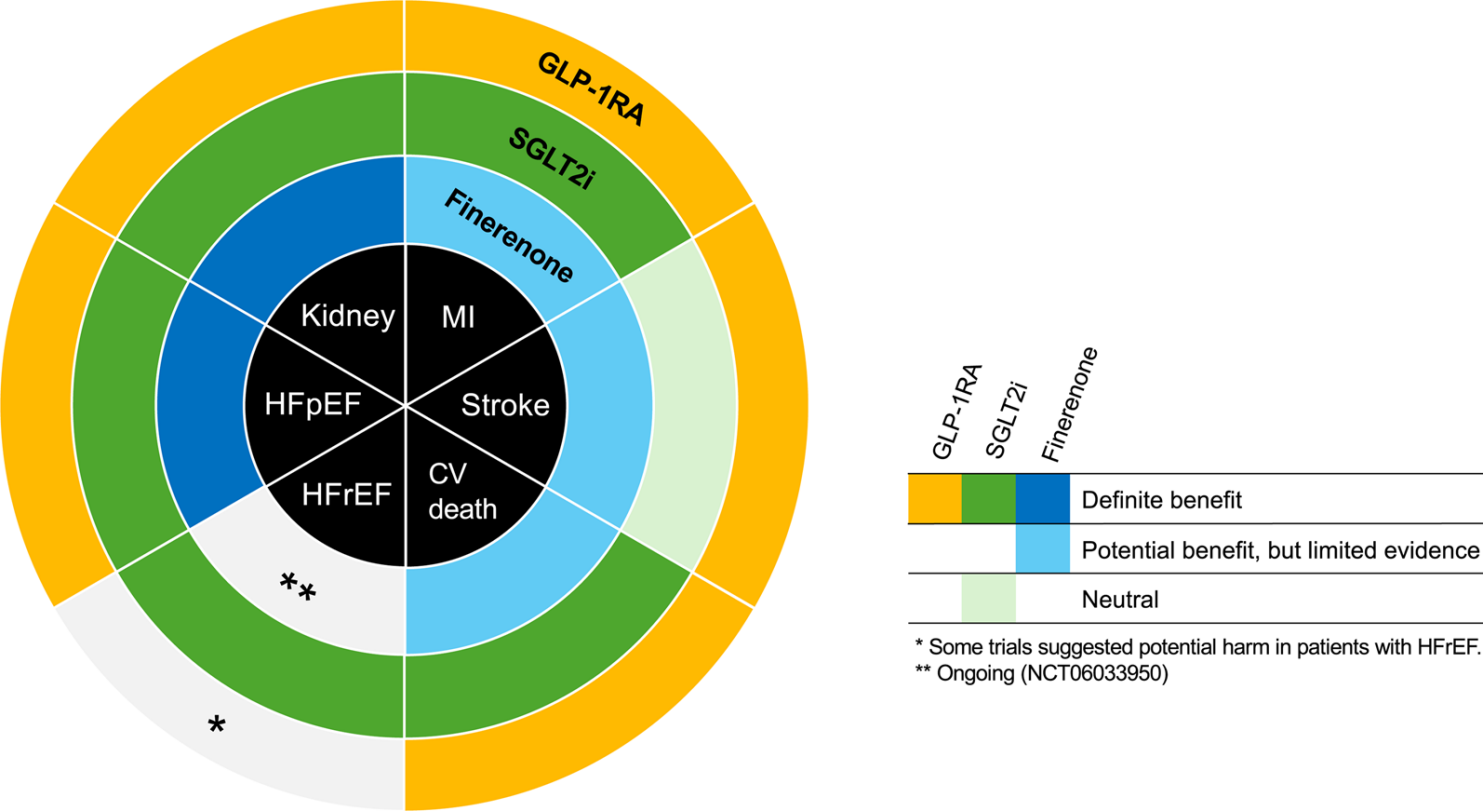

## Diabetes and risk of cardiovascular and kidney complications

Diabetes is one of the most important risk factors for cardiovascular disease (CVD) and chronic kidney disease (CKD). Compared with patients without diabetes, patients with diabetes have a 2–fourfold higher risk of atherosclerotic cardiovascular diseases (ASCVD), including coronary artery disease, stroke, and peripheral artery disease [1]. Moreover, fasting hyperglycaemia was responsible for 2.3 million cardiovascular deaths worldwide in 2021 [2]. Additionally, as the first CVD to develop in patients with diabetes is often heart failure rather than myocardial infarction [1], it is becoming clear that the pathology of diabetes itself causes heart failure through direct effects on cardiomyocytes and other myocardial cell types. CKD is a principal microvascular complication of diabetes and increases the risk of CVD and death [3]. About 40% of patients with diabetes could develop CKD [4], and it could be already present at diagnosis especially in patients with type 2 diabetes [5]. Furthermore, CKD in patients with diabetes is globally the leading cause of end-stage kidney disease and leading to increased health care cost due to renal replacement therapy [4].

Whether hyperglycaemia itself directly affects the pathogenesis of atherosclerosis and heart failure remains inconclusive; this is consistent with the fact that previous large clinical trials have not reached a conclusion on whether cardiovascular disease can be suppressed by strict glycaemic control [6]. Effect of glycaemic control itself on preventing CKD is relatively small, compared with multifactorial therapy in patients with diabetes [7, 8]. Lowering blood glucose levels is the basis of diabetes treatment, not only in terms of preventing microvascular complications, including CKD, but also in preventing cardiovascular events. In contrast, it is difficult to completely prevent the development of cardiovascular events and CKD by hypoglycaemic therapy alone. Thus, in addition to a comprehensive approach encompassing multidisciplinary risk factors (dyslipidaemia, hypertension, obesity, smoking, etc.) [8, 9], considering "what drugs to use to lower blood glucose" by selecting hypoglycaemic agents with evidence-based cardiorenal protective effects, according to patient background and risk status is increasingly important [10].

Glucagon-like peptide-1 receptor agonist (GLP-1RA) and sodium-glucose cotransporter 2 inhibitor (SGLT2i) are currently recommended as first-line hypoglycaemic agents for patients with diabetes with established cardiovascular disease or multiple cardiovascular and renal risks [10]. Particularly, GLP-1RAs have greater hypoglycaemic and weight-loss effects and are attracting attention as a means of intervening in obesity, which is the key basis of type 2 diabetes pathogenesis. The cardiorenal protective effects of finerenone, a novel nonsteroidal mineralocorticoid receptor antagonist (MRA), in patients with diabetes and CKD have also been revealed in recent years [11]. The strategies for preventing cardiovascular and renal events in patients with diabetes are becoming increasingly diverse. However, the results of clinical trials suggest that the characteristics of the cardiorenal protective effects achieved by these drugs differ slightly, and consensus for their use and evidence for their optimal use and combined benefits are still lacking. In this article, we primarily summarize the clinical effects of GLP-1RAs on the risk of cardiovascular and renal events and discuss the emerging role of their combined therapy with SGLT2is and finerenone.

## Mechanisms of CVD and CKD in patients with diabetes

Vascular damages of ASCVD in patients with diabetes are characterized by endothelial and vascular smooth muscle cell dysfunction [12]. Increased oxidative stress, inflammation and lipotoxicity associated with hyperglycaemia and insulin resistance potentially impair vascular endothelial function and increase endothelial permeability, leading to the penetration of glycated and oxidized low-density lipoprotein (LDL) cholesterol into the endothelium, i.e., initiation of atherosclerosis. Subsequently, macrophages transform into foam cells in the LDL cholesterol-deposited vascular endothelium. Hyperglycaemia has been suggested to cause increased expression of inflammation-related genes in macrophages. In vascular smooth muscle cells, hyperinsulinemia also promotes cell proliferation, contributing to the development of atherosclerosis and vascular remodelling. Independent of ASCVD and other risk factors, such as hypertension, diabetes also increases the risk of heart failure [13]. Myocardial remodelling characterized by fibrosis and hypertrophy, impaired microcirculation, abnormal protein function caused by advanced glycation end products, changes in myocardial metabolism and abnormal mitochondrial function have been noted as associated factors of diabetic cardiomyopathy [14].

The process of CKD progression in diabetes is also complicated [15]. Glomerular hyperfiltration due to hyperglycemia is one of the important features of CKD in diabetes and lead to diabetic glomerulopathy in combination with advanced glycation end products and inflammation. Inflammation and fibrosis can affect tubulo-interstitial lesions, and both glomerular and interstitial injuries will development of CKD. Comorbidities of diabetes (i.e., hypertension, dyslipidemia, obesity) also have crucial roles in pathophysiology of CKD in diabetes.

## GLP-1RA therapy on major adverse cardiovascular events

Representative large-scale cardiovascular and kidney outcome trials of GLP-1RAs and their results are shown in Table 1. The first study to demonstrate the effect of GLP-1RAs in reducing cardiovascular events was LEADER, in which 9340 patients with type 2 diabetes at high cardiovascular risk were randomized to liraglutide 1.8 mg per day or placebo [16]. The study showed a 13% reduction in major adverse cardiovascular events (MACE), a composite endpoint for cardiovascular death, nonfatal myocardial infarction, or nonfatal stroke, in patients treated with liraglutide compared with placebo at a mean follow-up of 3.8 years (hazard ratio [HR] 0.87, 95% confidence interval [CI] 0.78–0.97) and a significant reduction in cardiovascular death (HR 0.78, 95% CI 0.66–0.93). In SUSTAIN-6, wherein semaglutide (subcutaneous injection) was administered at 0.5 or 1 mg per week, MACE was also reduced by 26% in the semaglutide group compared with the placebo group (HR 0.74, 95% CI 0.58–0.95) as was stroke (HR 0.61, 95% CI 0.38–0.99) [17]. The risk of MACE was also significantly reduced in consecutive studies (Harmony Outcomes [18], REWIND [19], AMPLITUDE-O [20]). Their effects on the individual components of MACE, myocardial infarction (reduced in LEADER and Harmony Outcomes), and stroke (reduced in SUSTAIN-6 and REWIND), varied in each trial (Table 1). Although the reasons for the variation are still unclear, this might be due to several differences in the individual study design and patient characteristics rather than drug effect [21]. Moreover, in a meta-analysis of eight trials, GLP-1RAs reduced MACE by 14% (HR 0.86, 95% CI 0.79–0.94), cardiovascular death by 13% (HR 0.87, 95% CI 0.78–0.96), and stroke by 16% (HR 0.84, 95% CI 0.76–0.94) [22] (Table 2). Furthermore, in another meta-analysis [23], which excluded ELIXA [24] due to fact that lixisenatide is a short-acting GLP-1RA and all participants had acute coronary syndrome, GLP-1RAs were associated with significant reductions in MACE (HR 0.85, 95% CI 0.80–0.90), cardiovascular death (HR 0.85, 95% CI 0.78–0.93), stroke (HR 0.81, 95% CI 0.74–0.90), and myocardial infarction (HR 0.88, 95% CI 0.81–0.96) (Table 2).

**Table 1:** Summary of major large-scale clinical trials with GLP-1RAs

	ELIXA [24]	LEADER [16]	SUSTAIN-6 [17]	EXSCEL [36]	Harmony Outcomes [18]	REWIND [19]	PIONEER-6 [59]	AMPLITUDE-O [20]	SELECT [25]	FLOW [31]
Drug	Lixisenatide	Liraglutide	Semaglutide (s.c. injection)	Exenatide	Albiglutide	Dulaglutide	Semaglutide (oral)	Efpeglenatide	Semaglutide (s.c. injection)	Semaglutide (s.c. injection)
Dose	10 or 20 μg per day	1.8 mg per day	0.5 or 1.0 mg per week	2.0 mg per week	30 or 50 mg per week	1.5 mg per week	14 mg per day	4 or 6 mg per week	2.4 mg per week	1.0 mg per week
Number	6068	9340	3297	14,752	9463	9903	3183	4076	17,604	3533
Population	T2DM and history of acute coronary syndrome within 180 days	T2DM with high cardiovascular risk*	T2DM with high cardiovascular risk*	T2DM with or without previous CVD	T2DM with previous CVD	T2DM with previous CVD or risk factors of CVD	T2DM with high cardiovascular risk*	T2DM with previous CVD, or CKD and additional risk of CVD	BMI ≥ 27 kg/m^2^ and previous CVD without diabetes	T2DM and CKD
Observation period, years	2.1	3.8	3.1	3.2	1.6	5.4	1.3	1.8	3.3	3.4
Age, years	60	64	65	62	64	66	66	65	62	67
BMI, kg/m^2^	30.1	32.5	32.8	32.7	32.3	32.3	32.3	32.7	33.3	32.0
HbA1c, %	7.7	8.7	8.7	8	8.7	7.4	8.2	8.9	5.8	7.8
History of CVD, %	100	81	60	73.1	71	32	85	90	100	22.9
History of CHF, %	22	18	24	16.2	20	9	12	18	24	19.2
SGLT2i use at baseline, %	–	–	0.2	0.9 (ITT population)	6.1	–	10	15.2	–	15.6
MACE, HR (95% CI)	1.02** (0.89–1.17)	0.87 (0.78–0.97)	0.74 (0.58–0.95)	0.91 (0.83–1.00)	0.78 (0.68–0.90)	0.88 (0.79–0.99)	0.79 (0.57–1.11)	0.73 (0.58–0.92)	0.80 (0.72–0.90)	0.82 (0.68–0.98)
Cardiovascular death, HR (95% CI)	0.98 (0.78–1.22)	0.78 (0.66–0.93)	0.98 (0.65–1.48)	0.88 (0.76–1.02)	0.93 (0.73–1.19)	0.91 (0.78–1.06)	0.49 (0.27–0.92)	0.72 (0.50–1.03)	0.85 (0.71–1.01)	0.71 (0.56–0.89)
MI, HR (95% CI)	1.03 (0.87–1.22)	0.86 (0.73–1.00)	0.74 (0.51–1.08)	0.97 (0.85–1.10)	0.75 (0.61–0.90)	0.96 (0.79–1.15)	1.18 (0.73–1.90)	0.78 (0.55–1.10)	0.72 (0.61–0.85)	0.80 (0.55–1.15)
Stroke, HR (95% CI)	1.12 (0.79–1.58)	0.86 (0.71–1.06)	0.61 (0.38–0.99)	0.85 (0.70–1.03)	0.86 (0.66–1.14)	0.76 (0.61–0.95)	0.74 (0.35–1.57)	0.80 (0.48–1.31)	0.93 (0.74–1.15)	1.22 (0.84–1.77)
HHF, HR (95% CI)	0.96 (0.75–1.23)	0.87 (0.73–1.05)	1.11 (0.77–1.61)	0.94 (0.78–1.13)	0.85*** (0.70–1.04)	0.93 (0.77–1.12)	0.86 (0.48–1.55)	0.61 (0.38–0.98)	0.79 (0.60–1.13)	-
All cause death, HR (95% CI)	0.94 (0.78–1.13)	0.85 (0.74–0.97)	1.05 (0.74–1.50)	0.86 (0.77–0.97)	0.95 (0.79–1.16)	0.90 (0.80–1.01)	0.51 (0.31–0.84)	0.78 (0.58–1.06)	0.81 (0.71–0.93)	0.80 (0.67–0.95)
Composite kidney events, HR (95% CI)	–	0.78 (0.67–0.92)	0.64 (0.46–0.88)	–	–	0.85 (0.77–0.93)	–	0.68 (0.57–0.79)	0.78 (0.63–0.96)	0.76^****^ (0.66–0.88)

*BMI* body mass index, *CI* confidence interval, *CHF* congestive heart failure, *CVD* cardiovascular disease, *GLP-1RA* glucagon-like peptide-1 receptor agonist, *HHF* heart failure hospitalization, *HR* hazard ratio, *ITT* intention-to-treat, *MACE* major adverse cardiovascular events, *MI* myocardial infarction, *s.c.* subcutaneous, *SGLT2i* sodium-glucose cotransporter 2 inhibitor, *T2DM* type 2 diabetes mellitus

*Age of 50 years or more with at least one cardiovascular condition, or age of 60 years or more with at least one cardiovascular risk factor, ** 4-point MACE, *** Composite endpoint of CV death or HHF, **** Excluding macroalbuminuria

**Table 2:** Two meta-analyses of large GLP-1RA clinical trials involving patients with diabetes at high cardiovascular risk

	Giugliano et al. [22] 2021	Sattar et al. [23] 2021
MACE	0.86 (0.79–0.94)	0.85 (0.80–0.90)
Cardiovascular death	0.87 (0.78–0.96)	0.85 (0.78–0.93)
MI	0.91 (0.81–1.01)	0.88 (0.81–0.96)
Stroke	0.84 (0.76–0.94)	0.81 (0.74–0.90)
HHF	0.90 (0.83–0.98)	0.88 (0.79–0.98)
All cause death	0.88 (0.80–0.96)	0.87 (0.81–0.94)
Composite kidney events*	0.83 (0.73–0.94)	0.79 (0.73–0.87)

Data are presented as hazard ratio (95% confidence interval)

*GLP-1RA* glucagon-like peptide-1 receptor agonist, *HHF* heart failure hospitalization, *MACE* major adverse cardiovascular events, *MI* myocardial infarction

*Including macroalubuminuria

The results of SELECT, which examined the effect of subcutaneous semaglutide injection for the prevention of cardiovascular events in 17,604 patients with obesity (body mass index [BMI] ≥ 27 kg/m^2^) and established cardiovascular disease but without diabetes, were presented in 2023 [25]. During the mean observation period of 3.3 years, semaglutide at 2.4 mg per week resulted in a mean weight loss of − 9.39% from baseline and a 20% reduction in the risk of MACE (HR 0.80, 95% CI 0.72–0.90), especially a 28% reduction in the risk of myocardial infarction (HR 0.72, 95% CI 0.61–0.85), compared with that in the placebo group. GLP-1RAs not only have glucose-lowering but also greater weight-loss effects as discussed in detail in a previous literature [26], the results of SELECT also suggest the weight-loss effect as an important factor explaining the cardiovascular protective effects of GLP-1RAs regardless of diabetes. However, in Harmony Outcomes, albiglutide reduced the risk of MACE by 22%, with 0.52% and 0.83 kg reductions in HbA1c and weight, respectively, compared with those in the placebo group [18]. Factors independent of glycaemic and weight loss effects have also been suggested to affect the cardiovascular protective effects of GLP-1RAs.

## Heart failure therapy by GLP-1RAs

Although a meta-analysis of large-scale clinical trials suggested that GLP-1RAs reduce heart failure hospitalizations (HHF) by approximately 10% in patients with diabetes at high cardiovascular risk [22, 23], the treatment effects on the risk of HHF in individual trials other than AMPLITUDE-O were not significant (Tables 1, 2). Thus, unlike for MACE, the effect of GLP-1RAs in reducing the risk of HHF has not yet been consistently demonstrated in individual trials. FIGHT and LIVE were clinical trials involving the administration of liraglutide in patients with heart failure with reduced ejection fraction (HFrEF). Three hundred patients with HFrEF hospitalized for acute heart failure within 14 days and taking diuretics equivalent to at least 40 mg furosemide were randomized to receive 1.8 mg per day of liraglutide or placebo in FIGHT [27]. Their median left ventricular ejection fraction (LVEF) was 25%, 82% of the patients had ischaemic heart disease, and 59% had type 2 diabetes. At 180 days follow-up, death (HR 1.10, 95% CI 0.57–2.14) and heart failure rehospitalization (HR 1.30, 95% CI 0.89–1.88) did not differ compared with those in the placebo group. In addition, the results were similar for subgroup analysis by diabetes status. In LIVE, 241 clinically stable patients with HFrEF were randomized to receive liraglutide at 1.8 mg per day or placebo [28]. The mean LVEF was 33.7% and 35.4% in the liraglutide and placebo groups, respectively, and 60% of patients had ischaemic heart disease. During the 24-week observation, liraglutide did not improve LVEF but rather caused more death, ventricular arrhythmia, and atrial fibrillation, compared with the placebo. Thus, no evidence currently indicates that patients with HFrEF benefit from the administration of GLP-1RAs, but rather, the increased arrhythmias and death are concerning. One factor that may have contributed to such a result in patients with HFrEF is the increased heart rate caused by GLP-1RAs [21].

On the other hand, it is becoming clear that obesity and diabetes themselves affect the pathogenesis, progression, and prognosis of heart failure with preserved ejection fraction (HFpEF). The effect of semaglutide subcutaneous injection at 2.4 mg per week in patients with HFpEF and obesity (BMI ≥ 30 kg/m^2^) but without diabetes was tested in STEP-HFpEF [29]. The change in body weight from baseline was − 13.3% and − 2.6% in the semaglutide and placebo groups, respectively. The semaglutide group showed a significantly improved Kansas City Cardiomyopathy Questionnaire clinical summary score and a longer 6-min walking distance from baseline (21.5 m and 1.2 m in the semaglutide and placebo groups, respectively, intergroup difference 20.3 m, 95% CI 8.6–32.1 m), compared with that in the placebo group despite the relatively short observation period of 52 weeks. Similar results were reported in STEP-HFpEF DM, which included patients with diabetes excluded in STEP-HFpEF [30]. Pooled analysis including these two studies in addition to SELECT and FLOW [31] reported that semaglutide reduced the risk of hospitalization or urgent visits due to heart failure by 41% (HR 0.59, 95% CI 0.41–0.82) in HFpEF patients compared with placebo [32]. In contrast, the effect on cardiovascular death did not reach at the statistical significance (HR 0.82, 95% CI 0.57–1.16), possibly due to lower event rate. Nearly all patients were overweight or obesity (BMI ≥ 27 kg/m^2^) in this analysis, and reduction of worsening heart failure event was more compelling in patients with higher BMI (HR 0.49, 95% CI 0.33–0.70 in BMI ≥ 35 kg/m^2^, HR 0.96 95% CI 0.67–1.38 in BMI < 35 kg/m^2^). Weight loss effect of semaglutide could be one of important factors leading to improved quality of life in patients with HFpEF and overweight. However, the effect beyond weight loss was also indicated by decreasing trend of NT-proBNP or C-reactive protein levels independent of weight loss in STEP-HFpEF [33, 34].

Collectively, semaglutide improved the heart failure-related symptoms and reduce the incidence of worsening heart failure events especially in HFpEF patients with higher BMI, suggesting that it could be a promising therapeutic approach for this patient population. However, whether this could be a class effect of GLP-1RA remains uncertain, and the evidence on HFrEF is scarce.

## GLP-1RA therapy on kidney outcomes

In individual clinical trials of GLP-1RAs, the therapy improved the kidney outcomes compared with those of placebo treatment, although most of these results emanated from secondary outcomes or exploratory analysis. Zelniker et al. [35] defined kidney outcomes as broad (new onset of macroalbuminuria, worsening estimated glomerular filtration rate [eGFR], end-stage kidney disease, or death attributable to renal causes) and narrow composite (excluding macroalbuminuria from a broad composite) and performed a meta-analysis of ELIXA, LEADER, SUSTAIN-6, and EXSCEL [36]. Broad composite outcomes were significantly reduced compared with those following placebo treatment (HR 0.82, 95% CI 0.75–0.89), but no statistically significant reduction in narrow composite outcomes, except for macroalbuminuria, was observed (HR 0.92, 95% CI 0.80–1.06). In a meta-analysis by Sattar et al., composite kidney outcomes, including macroalbuminuria, were reduced by 21% compared with those in the placebo group (HR 0.79, 95% CI 0.73–0.87). Although the incidence of worsening kidney function outcome based predominantly on eGFR change was nominally reduced after removal of ELIXA (0.82, HR 95% CI 0.69–0.98), composite outcomes excluding macroalbuminuria, mainly declining eGFR and doubling creatinine, were not significant (HR 0.86, 95% CI 0.72–1.02) [23]. Therefore, most of renoprotective effects of GLP-1RAs were likely to be due to the inhibition of progression to macroalbuminuria [35], and the effects on other clinically important renal endpoints, including eGFR decline, initiation of renal replacement therapy and renal death, was unclear [5].

The result of FLOW, a study to assess the effect of subcutaneous semaglutide injection on CKD in patients with type2 diabetes, was reported recently [31]. In this study, 3533 patients with CKD (eGFR of 50–75 ml/min/1.73 m^2^ and a UACR of > 300 and < 5000 mg/gCr or an eGFR of 25 to < 50 ml/min/1.73 m^2^ and a UACR of > 100 and < 5000 mg/gCr) and diabetes were enrolled and assigned to 1 mg per week subcutaneous semaglutide injection or a placebo group. The primary outcome defined as time to first kidney failure (persistent eGFR < 15 mL/min/1.73 m^2^ or initiation of chronic renal replacement therapy), ≥ 50% eGFR decline from baseline, or death from renal or cardiovascular causes was lower in semaglutide group (HR 0.76, 95% CI 0.66–0.88). In the component of primary outcome, persistent ≥ 50% eGFR decline from baseline was significantly reduced in semaglutide group (HR 0.73, 95% CI 0.59–0.89). Initiation of renal replacement therapy tended to decrease, but did not reach to statistical significance (HR 0.84, 95% CI 0.63–1.12). Thus, this study firstly added an insight that semaglutide can favourably affect more clinically important renal endpoints in patients with type 2 diabetes and CKD.

## Potential mechanisms underlying clinical benefits of GLP-1RAs

GLP-1 receptors are thought to be expressed on atrial and ventricular cardiomyocytes, endothelial cells in the human heart, and endothelial and smooth muscle cells in blood vessels [21]. However, humans and mice display large differences. Furthermore, information on GLP-1 receptor expression in the heart is still lacking, and whether GLP-1RAs act directly or indirectly on the cardiovascular system remains inconclusive. Nevertheless, in vitro studies on blood vessels have shown that GLP-1RAs inhibit vascular smooth muscle cell proliferation, reduce reactive oxygen species, and increase nitric oxide in vascular endothelial cells [37].

In animal models of heart failure induced by high-frequency pacing, ischaemia, and obesity, GLP-1RAs inhibit left ventricular remodelling and improve left ventricular contractility and diastolic function through anti-inflammatory effects, cardiomyocyte apoptosis inhibition, and glucose metabolism enhancement in cardiomyocytes [38–40]. However, in a LIVE sub-study, myocardial glucose utilization did not improve in the liraglutide group as examined by F-fluorodeoxyglucose positron emission tomography [41], similar to a study using albiglutide [42]. This discrepancy between results in animal models and clinical studies may partly be due to myocardial insulin resistance in severely failing hearts and the use of beta-blockers [21].

Also in kidney, localization of GLP-1 receptor is under investigation, but it is thought to be expressed in vascular smooth muscle cells and immune cells in kidney [43]. Although GLP-1RAs have inhibitory effect on sodium hydrogen exchanger-3 in tubules, it is unclear that renoprotective effect of GLP-1RAs is attributable to hemodynamic effect [44]. Preclinical studies suggested that GLP-1RAs reduced immune cell activity and oxidative stress in kidney and led to decrease in fibrosis [15, 45]. Immunomodulation effects are currently thought to have principal roles in renoprotective effect of GLP-1RAs.

## Potential complementary evidence of GLP-1RAs and SGLT2is

In a meta-analysis comparing the effects of GLP-1RAs with SGLT2is, five GLP-1RA (ELIXA, LEADER, SUSTAIN-6, EXSCEL, and Harmony Outcomes) and three SGLT2i trials (EMPA-REG OUTCOME, CANVAS Program, and DECLARE-TIMI 58) with a total of 77,242 patients were include [35]. The risk of MACE was reduced by 12% (HR 0.88, 95% CI 0.84–0.94) with GLP-1RAs and by 11% (HR 0.89, 95% CI 0.83–0.96) with SGLT2is compared with that in placebo groups, suggesting that GLP-1RAs and SGLT2is reduce the risk of MACE to a similar extent. Furthermore, the risks of myocardial infarction (HR 0.91, 95% CI 0.84–0.98 with GLP-1RAs and HR 0.89, 95% CI 0.80–0.98 with SGLT2is) and cardiovascular death (HR 0.88, 95% CI 0.80–0.96 with GLP-1RAs and HR 0.84, 95% CI 0.75–0.94 with SGLT2is) were similarly reduced in both GLP-1RA and SGLT2i groups, while the risk of stroke was reduced only with GLP-1RAs (HR 0.86, 95% CI 0.77–0.97 with GLP-1RAs and HR 0.97, 95% CI 0.86–1.10 with SGLT2is). On the other hand, the risks of HHF (HR 0.93, 95% CI 0.83–1.04 with GLP-1RAs and HR 0.69, 95% CI 0.61–0.79 with SGLT2is) and composite kidney outcomes excluding macroalbuminuria (HR 0.92, 95% CI 0.80–1.06 with GLP-1RAs and HR 0.55, 95% CI 0.48–0.64 with SGLT2is) significantly reduced with SGLT2is only. Particularly for heart failure, several clinical trials and meta-analyses have already confirmed that SGLT2is reduce HHF by approximately 30% in both HFrEF and HFpEF, with or without diabetes [46]. Thus, the clinical benefits for the risk of heart failure and kidney outcomes, except albuminuria, are likely to be a strength of SGLT2is, whereas those for the risk of stroke are a strength of GLP-1RAs (Graphical Abstract).

It may be reasonable to focus on whether the combination of GLP-1RAs and SGLT2is, which are expected to have complementary effects based on large-scale clinical trial results, can further reduce cardiovascular and renal events in patients with diabetes. However, currently, no trial has directly examined the effects of this combination therapy. In previous large-scale clinical trials of GLP-1RAs, 15.2% (*n* = 618) of AMPLITUDE-O and 6.1% (*n* = 575) of Harmony Outcomes patients used SGLT2is at baseline [18, 20] (Table 1). In the AMPLITUDE-O *post-hoc* analysis, the HR for MACE was 0.70 (95% CI 0.37–1.30) with baseline SGLT2is and 0.74 (95% CI 0.58–0.94) without SGLT2is (*P*-value for interaction 0.70), and the HR for HHF was 0.23 (95% CI 0.05–0.97) with SGLT2is and 0.70 (95% CI 0.42–1.17) without SGLT2is (*P*-value for interaction 0.35) [47]. For composite kidney outcomes, the HR was 0.52 (95% CI 0.33–0.83) with SGLT2is and 0.70 (95% CI 0.59–0.83) without SGLT2is (*P*-value for interaction 0.38) (Table 3). Therefore, considering cardiorenal protective effects, no interaction was observed between baseline SGLT2i use and efpeglenatide. The Harmony Outcomes *post-hoc* analysis also showed a similar trend with respect to MACE and HHF, although kidney outcomes were not examined (Table 3) [48]. No difference in adverse events was observed with the SGLT2i combination in the respective Harmony Outcomes and AMPLITUDE-O *post-hoc* analyses. In addition, a meta-analysis of 1193 patients with baseline SGLT2i use in AMPLITUDE-O and Harmony Outcomes revealed an HR for MACE of 0.78 (95% CI 0.49–1.24) with SGLT2is and 0.77 (95% CI 0.68–0.87) without SGLT2is (*P*-value for interaction 0.95), and an HR for HHF of 0.34 (95% CI 0.12–0.96) with SGLT2is and 0.72 (95% CI 0.55–0.92) without SGLT2is (*P*-value for interaction 0.18) [48]. Moreover, a similar trend has been observed in the *post-hoc* analysis of clinical trials for SGLT2is. In DECLARE-TIMI58 with dapagliflozin, 4.4% of the patients were using GLP-1RAs at baseline, and dapagliflozin reduced the risks of MACE, HHF, and composite kidney outcomes regardless of baseline GLP-1RA use [49]. Specifically, the HR for HHF was 0.20 (95% CI 0.07–0.60) with GLP-1RAs and 0.77 (95% CI 0.64–0.92) without GLP-1RAs (*P*-value for interaction 0.014). Although the CIs were wide because of the small number of events in subgroups, point estimates showed that the combination of GLP-1RAs and SGLT2is tended to reduce the incidence of HHF more than either alone. Additive effect on kidney outcomes including macroalbuminuria was also expected from AMPLITUDE-O substudy, however, additive effect of semaglutide and SGLT2i on the composite kidney outcomes without macroalbuminuria was not indicated in the FLOW substudy [50]. This may partly because SGLT2i users at baseline were few (15.6%) and renal events occurred later than MACE in the FLOW. The mechanisms of action of GLP-1RA and SGLT2i are essentially independent [51], and further studies are therefore warranted to assess whether their combination therapy can provide greater clinical benefit.

**Table 3:** Impact of GLP-1RAs on clinical outcomes according to baseline combination use with SGLT2is

Outcomes	Baseline SGLT2i Use	Individual Trials		Meta-analysis [48]
		AMPLITUDE-O [47] (Epfegrenatide)	Harmony Outcomes [48] (Albiglutide)	
MACE	With SGLT2i	0.70 (0.37–1.30)	0.89 (0.45–1.77)	0.78 (0.49–1.24)
	Without SGLT2i	0.74 (0.58–0.94)	0.78 (0.67–0.90)	0.77 (0.68–0.87)
HHF	With SGLT2i	0.23 (0.05–0.97)	0.50 (0.12–2.08)	0.34 (0.12–0.96)
	Without SGLT2i	0.70 (0.42–1.17)	0.72 (0.54–0.97)	0.72 (0.55–0.92)
Composite kidney events*	With SGLT2i	0.52 (0.33–0.83)	N/A	N/A
	Without SGLT2i	0.70 (0.59–0.83)	N/A	N/A

Data are presented as hazard ratio (95% confidence interval)

*GLP-1RA* glucagon-like peptide-1 receptor agonist, *HHF* heart failure hospitalization, *MACE* major adverse cardiovascular events, *N/A* not available, *SGLT2i* sodium-glucose cotransporter 2 inhibitor

*Including macroalubuminuria

## Combination with finerenone for cardiorenal protection

Recently, in FIDELIO-DKD [52], FIGARO-DKD [53] and their pooled meta-analysis FIDELITY [11], finerenone has also been shown to reduce cardiovascular and renal events in CKD patients with diabetes. The cardiovascular benefit of finerenone in CKD patients with diabetes was primarily attributable to the reduction in the risk of HHF and was similar in FINEARTS-HF for patients with HFpEF [54]. In the FINE-HEART pooled analysis of these three large clinical trials, finerenone also reduced the risks of HHF, composite kidney outcome without macroalbuminuria, MACE, and all cause death [55]. Among participants, background use of GLP-1RA and SGLT2i was 5.8% and 8.9%, respectively, and treatment effect of finerenone on cardiovascular death was consistent regardless of those medication usages. The clinical benefits of finerenone on cardiovascular and kidney events are also unlikely affected by combination therapy with GLP-1RA or SGLT2i, however, clinical evidence on additive effects of finerenone in combination with GLP-1RA or SGLT2i are still limited [56, 57]. The CONFIDENCE is currently examining the effect of empagliflozin and finerenone combination therapy on kidney outcomes in CKD patients with diabetes [58].

## Triple combination therapy

Neuen et al. performed a cross-trial analysis using data from clinical trials of GLP-1RAs (ELIXA, LEADER, SUSTAIN-6, EXSCEL, HARMONY, REWIND, PIONEER-6 [59], and AMPLITUDE-O), SGLT2is (CANVAS and CREDENCE), and finerenone (FIDELIO-DKD and FIGARO-DKD) to estimate the effects of the triple combination therapy of GLP-1RA, SGLT2i, and finerenone using the actuarial method [60]. In patients with diabetes and albuminuria, the HRs for MACE, HHF, and CKD progression with triple combination therapy were estimated to be 0.65 (95% CI 0.55–0.76), 0.45 (95% CI 0.34–0.58), and 0.42 (95% CI 0.31–0.56), respectively, compared with those for conventional therapy alone (renin-angiotensin system inhibitors and classical risk factor management). The mechanisms of action of GLP-1RAs, SGLT2is, and nonsteroidal MRA are generally considered complementary and independent but may overlap in part, and the effects of each drug may not be completely additive when the three drugs are combined. Therefore, when estimated assuming 50% additivity, the HRs were 0.73 (95% CI 0.62–0.87) for MACE, 0.53 (95% CI, 0.41–0.70) for HHF, and 0.51 (95% CI, 0.38–0.68) for CKD progression. Some attenuation in HR was observed, but the impact of triple combination therapy on MACE, HHF, and CKD progression was still consistent, assuming 50% additivity. When patients aged 50 years old start triple therapy, event-free survival was estimated to increase by 3.2 years (95% CI 2.1–4.3 years) for MACE, 3.2 years (95% CI 2.4–4.0 years) for HHF, and 5.5 years (95% CI 4.0–6.7 years) for CKD progression compared with that for conventional therapy alone. Results of this integrated analysis suggest that the triple combination of GLP-1RA, SGLT2i, and finerenone provides stronger cardiorenal protective benefits than either conventional therapy or each alone. Given existing evidence, triple combination therapy can be expected to provide additional cardiorenal protection, although further randomized controlled trials are needed to determine whether triple combination therapy actually has additive or synergetic effects compared with mono or dual therapy.

## Summary and future perspectives of GLP-1RA therapy

Thus, we have currently obtained multiple therapeutic tools that reduce the risks of cardiovascular and renal events in patients with diabetes. Moreover, their combined use has the potential to further enhance treatment effects. Appropriate drug choice according to the patient clinical situation will lead to the precision medicine in the field of cardiovascular-kidney-metabolic syndrome.

Given the clinical evidence reviewed above, patients with type 2 diabetes and/or obesity are the most promising candidate population to benefit from GLP-1RA-centered pharmacotherapy to improve cardiovascular and kidney outcomes. GLP-1RAs have reliable hypoglycaemic and weight-loss effects that can intervene in obesity—the basis of type 2 diabetes pathology—and multifaceted effects on other cardiometabolic parameters, such as blood pressure and circulating lipids [21]. Nevertheless, since the risk of cardiovascular and renal events will remain even with the use of GLP-1RA, there is a great deal of clinical interest in its combined use with SGLT2i and finerenone (Table 4). These agents potentially have complementary effects in cardiovascular and renal protection (Graphical Abstract).

**Table 4:** Pros and evidence gaps of combination therapy

Pros	Evidence gaps
Multifactorial favorable effects on cardiometabolic parameters, including glycemia, body weight, blood pressure, and lipid profiles Additive/synergetic cardiovascular and renal benefits, as inferred from clinical trials and mechanistical insights Complementary mechanism of action on residual cardiovascular and renal risks	Need to identify the suitable patient population who better merit from the combination therapy When and how to implement in the clinical settings Long-term adherence and safety Cost-effectiveness

To further implement GLP-1RA-centric combination therapy, we need to consider some evidence gaps (Table 4). First, we should identify populations that are suitable or unsuitable for GLP-1RA-centric combination therapy. In recent GLP-1RA trials focusing on patients with obesity, favourable effect on worsening heart failure in HFpEF patients was indicated to be modulated by baseline BMI [32], although the underlying mechanisms that explain the cardiovascular and renal benefits may not necessarily be due to weight loss alone. Since background BMI can be largely different between races, caution is needed when interpreting and applying the results, including combination use with SGLT2is and finerenone. In addition, few studies currently support the use of GLP-1RAs in patients with HFrEF. Second, determining the ideal timing and priority to intensify therapy is also urgently required, since present evidence are not enough to cover this evidence gap. In particular, treatment effect size may differ among each combination and be affected by patient clinical situations and targeting outcomes. Third, long-term adherence and safety of the combination therapy need to be further addressed. Finally, cost-effectiveness is an important issue that is often addressed in multidrug regimen research. Given the tremendous costs of treating cardiovascular disease and continuing renal replacement therapy, the suspected total benefits obtained from therapy should not be overlooked. Therefore, continuous assessment is also required to determine the cost-effectiveness of the combination therapy.

## Data Availability

No datasets were generated or analysed during the current study.

## Abbreviations

ASCVD: Atherosclerotic cardiovascular disease
BMI: Body mass index
CI: Confidence interval
CKD: Chronic kidney disease
CVD: Cardiovascular disease
eGFR: Estimated glomerular filtration ratio
GLP-1RA: Glucagon-like peptide-1 receptor agonist
HFpEF: Heart failure with preserved ejection fraction
HFrEF: Heart failure with reduced ejection fraction
HHF: Heart failure hospitalization
HR: Hazard ratio
LDL: Low-density lipoprotein
LVEF: Left ventricular ejection fraction
MACE: Major cardiovascular events
MRA: Mineralocorticoid receptor antagonist
SGLT2: Sodium-glucose cotransporter 2
